# Prenatal Exposure to a Common Organophosphate Insecticide Delays Motor Development in a Mouse Model of Idiopathic Autism

**DOI:** 10.1371/journal.pone.0121663

**Published:** 2015-03-24

**Authors:** Alessia De Felice, Maria Luisa Scattoni, Laura Ricceri, Gemma Calamandrei

**Affiliations:** 1 Section of Neurotoxicology and Neuroendocrinology, Department of Cell Biology and Neurosciences, Istituto Superiore di Sanità, Rome, Italy; 2 Department of Physiology and Pharmacology “Vittorio Erspamer”, Sapienza University of Rome, Rome, Italy; Universidade do Estado do Rio de Janeiro, BRAZIL

## Abstract

Autism spectrum disorders are characterized by impaired social and communicative skills and repetitive behaviors. Emerging evidence supported the hypothesis that these neurodevelopmental disorders may result from a combination of genetic susceptibility and exposure to environmental toxins in early developmental phases. This study assessed the effects of prenatal exposure to chlorpyrifos (CPF), a widely diffused organophosphate insecticide endowed with developmental neurotoxicity at sub-toxic doses, in the BTBR T+tf/J mouse strain, a validated model of idiopathic autism that displays several behavioral traits relevant to the autism spectrum. To this aim, pregnant BTBR mice were administered from gestational day 14 to 17 with either vehicle or CPF at a dose of 6 mg/kg/bw by oral gavages. Offspring of both sexes underwent assessment of early developmental milestones, including somatic growth, motor behavior and ultrasound vocalization. To evaluate the potential long-term effects of CPF, two different social behavior patterns typically altered in the BTBR strain (free social interaction with a same-sex companion in females, or interaction with a sexually receptive female in males) were also examined in the two sexes at adulthood. Our findings indicate significant effects of CPF on somatic growth and neonatal motor patterns. CPF treated pups showed reduced weight gain, delayed motor maturation (i.e., persistency of immature patterns such as pivoting at the expenses of coordinated locomotion) and a trend to enhanced ultrasound vocalization. At adulthood, CPF associated alterations were found in males only: the altered pattern of investigation of a sexual partner, previously described in BTBR mice, was enhanced in CPF males, and associated to increased ultrasonic vocalization rate. These findings strengthen the need of future studies to evaluate the role of environmental chemicals in the etiology of neurodevelopment disorders.

## Introduction

Autism spectrum disorders (ASD) are characterized by marked reduction of social and communicative skills and presence of stereotyped movements associated with restricted interests. The etiological bases of this group of disorders are still largely unexplained. Susceptibility to ASD is attributable to both genetic and environmental factors [1, 2]: recent studies of ASD concordance in monozygotic and dizygotic twins suggest a moderate genetic heritability and a substantial environmental component [3]. There is general agreement that ASD may result from the complex interaction between multiple genes conferring vulnerability and diverse environmental factors, including early prenatal exposure to environmental contaminants [4], maternal infection during pregnancy [5–8], or advanced parental age [9]. Notably perinatal exposure to widespread environmental pollutants (i.e. heavy metals, organophosphate pesticides) appears to increase the risk of ASD but may not be sufficient *per se* to cause ASD [10–13]. If both genes and environment converge, a resulting dysfunction of neurotransmitters and signalling pathways could take place at key developmental time points [14].

Rodent models are important tools for understanding how genetic variation and exposure to toxic agents may interact in causing altered neurodevelopment. Although several mouse models bearing mutations in ASD candidate genes are available to date, only few studies have evaluated the contribution of environmental neurotoxicants to the neurobehavioral phenotype in selected mutant mouse strains [15, 16].

In this study we examined the effects of gestational exposure to a widely diffused neurotoxicant, the organophosphate insecticide chlorpyrifos (CPF), in a validated mouse model of idiopathic autism, the BTBR T+tf/J mice. BTBR T+tf/J (BTBR) is an inbred mouse strain that displays several behavioral traits relevant to autism, including impairments in social and communication domains [17–21], as shown by alterations in emission of ultrasonic vocalizations (USVs) [22–25] and high levels of repetitive behaviors. The inherited genetic changes that lead to autistic-like behaviors in these mice are incompletely known and still under active investigation [26]. Unlike transgenic knockout mouse models, whose altered phenotype may be causally related to diminished or absent expression of single major genes, the impaired sociability of BTBR mice may reflect subtle epistatic interactions within a network of related genes, many of which may be normal polymorphisms [27, 28].

The behavioral ontogeny of BTBR mice has been described by Scattoni and co-workers: they showed that BTBR pups reached some developmental milestones earlier than the C57BL/6J mouse strain, presenting shorter latencies in righting reflex response and quicker orientation towards olfactory stimuli in the homing test [22]. However, the same authors also showed abnormalities in vocalization patterns of BTBR pups separated from their mothers and siblings, indicative of a potential communication deficit during early stages of ontogeny. In particular, BTBR mouse pups show higher vocalization rates in comparison to other strains of mice and emit a restricted repertoire of calls as defined by their sonographic patterns [22]. At adulthood BTBR mice have lower level of social investigation in all social contexts examined in comparison to the highly sociable C57BL/6J strain. Specifically, BTBR adult mice display less anogenital sniffing and fewer ultrasonic vocalizations than C57BL/6J while approaching a same- or different-sex companion [23], and they show reduced motivation to interact with relevant social cues [29], poor behavioral flexibility in learning tasks [19, 30, 31] and stereotyped self-grooming behavior [18, 30, 32, 33]. Altogether, the neurobehavioral characteristics of the BTBR mice make this strain a good candidate to test the hypothesis of higher susceptibility of ASD individuals to the effects of neurotoxic compounds in critical developmental stages [13].

As a developmental neurotoxicant we selected chlorpyrifos (CPF), one of the most diffused non-persistent organophosphate (OP) insecticides worldwide [34], whose acute neurotoxic effects reside on inhibition of brain acetylcholinesterase (AChE) activity. Reasons for choosing CPF as an environmental chemical to be tested in this strain of mice are twofold. First, OPs and CPF in particular have recently been listed among the environmental chemicals possibly responsible for increased ASD risk [4]. Specifically, several epidemiological studies indicate that environmentally relevant exposure *in utero* to CPF and other OPs may affect children’s neuropsychological maturation, in terms of impaired reflex functioning in new borns [35, 36] and decreased mental and psychomotor performances and attention problems in infants, with higher risk to develop pervasive developmental disorders [37].

Secondly, an increasing body of experimental rodent data indicates that, at subtoxic doses, developmental exposure to CPF affects neurobehavioral maturation, targeting neural and neuroendocrine systems involved in ASD etiology [38–42], and causing multiple behavioral alterations in rats and mice at the neonatal, juvenile and adult stage as for motor activity, spatial learning and social responses. Specifically, in juvenile and adult CD-1 out bred mice, we described several changes in response to socially relevant cues, including enhanced agonistic responses and hyperactivity in males, increased social investigation in females, and altered pattern of investigation of either familiar or unfamiliar individuals in both sexes [43, 44]. Limited effects on development of motor coordination skills have been described in rats after neonatal exposure to CPF [45], while in CD-1 mice gestational treatment with CPF induces small alterations at the neonatal stage, such as hyporeflexia and decrease in the emission of ultrasonic vocalizations at postnatal day 10 in the exposed offspring [46].

In the present study we administered pregnant mice of the BTBR inbred strain with vehicle or CPF at the sub-toxic dose of 6 mg/kg/bw by oral gavage from gestational day (GD) 14 to 17, replicating as for dose and administration schedule our previous studies carried out in the CD-1 outbred strain [46]. Our main aim was to evaluate in the offspring of both sexes the effect of the gestational CPF exposure on spontaneous locomotion, ultrasonic vocalization and neurodevelopment during the first two weeks of postnatal life, to evidence early changes in behavioral profile. Our hypothesis was that CPF might enhance the developmental abnormalities previously described by our research group in BTBR pups (enhanced rate of vocalization and restricted repertoire of calls) and possibly affect the ontogeny of neonatal motor behavior patterns so far never analyzed in depth in this mouse strain.

We show here significant effects of CPF on somatic growth and early neonatal motor patterns, with persistency of immature motor patterns such as pivoting at the expenses of coordinated locomotion, but no significant effects on ultrasonic calling rate and call categories.

On the basis of the results obtained in the neonatal stage we decided to evaluate the long-term effects of CPF on selected markers of the peculiar behavioral repertoire of this mouse strain, namely investigative response to same-sex individuals in females, and approach to a sexual partner in males. At adulthood, limited CPF associated alterations were found in males only: the abnormal pattern of social investigation of the receptive female reported for BTBR mice was amplified in CPF males and associated to increased ultrasonic vocalization rate. The high rate of self-grooming expressed by BTBR mice was not significantly influenced by CPF treatment. These findings only partially support the hypothesis that CPF worsens the behavioral phenotype of BTBR mice, but highlights a specific vulnerability to CPF effects as for early motor development not previously evidenced in other mouse strains.

## Materials and Methods

### Ethics statement

This study was carried out in accordance with the Italian Animal Welfare legislation (art. 4 and 5 of D.L. 116/92) that implemented the European Committee Council Directive (2010/63/EEC). The Italian Ministry of Health specifically approved the protocol of this study on 10/31/2011, Authorization n° 223/2011-B to G.C.

### Animals

Male and female mice of the BTBR T+tf/J strain purchased from the Jackson Laboratory (Bar Harbour, ME, USA) were housed upon the arrival in breeding cages (polycarbonate cages 33x13x14 cm) under standard animal housing conditions (temperature 20 ± 2°C; humidity 60–70%) with food (enrichment standard diet for mice from Altromin, Spezialfutter GmbH & Co. Germany) and water ad libitum, under a 12:12 reverse light cycle (lights on from 8:00 p.m. till 8:00 a.m.).

Females were inspected daily for the presence of the vaginal plug (GD 0). On GD 14, pregnant females were randomly assigned to one of the two prenatal treatments [vehicle (Veh), CPF]. CPF (Chem. Service, West Chester, PA) was dissolved in peanut oil (Veh) to provide rapid and complete absorption. CPF (in a volume of 0.1 ml/10 g at a dose of 6 mg/kg/bw) or its vehicle was administered to pregnant females from GD 14 to 17 by intraoral gavage. Due to the lower reproductive efficiency of the BTBR strain in comparison to other outbred and inbred mouse strains currently used in toxicological studies, we selected a single dose level among those already assessed in previous multidose studies performed in our laboratory. Specifically, we have shown that CPF at doses ranging from 1 to 6 mg/kg/bw, and administered by different routes either on GD 14–17 and/or directly to pups in the first two weeks of postnatal life, induced several behavioral changes in the offspring, but the late gestational exposure window and the 6 mg/kg dose was those producing the most significant effects on social responses [43, 46]. The dose selected is safe with respect to reproductive performance of treated dams (pregnancy length, number of pups at delivery, sex ratio), and it does not induce systemic toxicity in dams or major effects on pup’s health such as weight at delivery and growing rate [47]. Furthermore, this same CPF treatment schedule and dose does not affect brain AChE activity in offspring when measured 24 hrs following the last CPF exposure, but causes only a mild transient inhibition (20% of control values) in serum AChE activity at birth [47]. Recently, Moreira and coworkers [48] found a dose-dependent effects on AChE inhibition in C57BL/6 mice, with a LOAEL for maternal brain at 10 mg/kg CPF and with up to a 60% inhibition in the 15 mg/kg, while in the fetal brain significant inhibition occurred only in the 10 mg/kg dose group (35% inhibition). These findings indicate that the effects of CPF on AChE inhibition in the fetal brain following gestational exposure are comparable in out bred and inbred mouse strains, and that the dose used in the present study is under the threshold of systemic toxicity.

Twenty-four litters (13 Vehicle-treated and 11 CPF-treated) were used. Females’ body weight was monitored daily during pregnancy. Proportion of term pregnancies, gestation length, litter size, sex ratio and neonatal mortality were also measured to exclude potential effects of the treatment on reproductive performances. The day of birth was defined as postnatal day 0 (pnd 0). On the day of birth, the sex of the pups was assessed by evaluation of anogenital distance. Body weight of each individual pup was recorded at birth, on each of the four days of neonatal assessment, at weaning (day 21) and at adulthood (at the time of social behavior assessment). For identification purposes, on pnd 4 pups were tattooed on the paw with animal tattoo ink (Ketchum permanent Tattoo Inks green paste, Ketchum Manufacturing Inc., Brockville ON Canada). All behavioral procedures were carried out during the dark phase of the cycle between 10:00 a.m. and 2:00 p.m. under dim lights.

### Behavioral assessment in the neonatal stage

#### Recording of ultrasonic vocalizations in separated pups

USVs are an important tool to assess emotional development and communication between mother and infants, as they elicit pup retrieval by the parents and maternal licking [49, 50]. One female and one male offspring from each litter of either Veh- (n = 13) or CPF-treated (n = 11) BTBR dams were used for evaluation of ultrasonic calls emitted on pnd 4, 6, 8 and 12. On each day of testing, a single pup was placed into an empty glass container (diameter 5 cm; height 10 cm), located inside a sound-attenuating Styrofoam box, and USVs were assessed during a 3-min test. At the end of the 3-min recording session, each pup was weighed and its axillary temperature measured by gentle insertion of the thermometer tip in the skin pocket between upper forelimb and chest of the animal for about 5 seconds.

An Ultrasound Microphone (Avisoft Ultrasound Gate condenser microphone capsule CM16, Avisoft Bioacoustics, Berlin, Germany) sensitive to frequencies of 10–180 kHz recorded the pup vocalizations in the sound-attenuating chamber. The microphone was placed through a hole in the middle of the cover of the Styrofoam sound-attenuating box, about 20 cm above the pup. The temperature of the room was maintained at 22 ± 1°C. Vocalizations were recorded using Avisoft Recorder software (Version 3.2). Settings included sampling rate at 250 kHz and format 16 bit. For acoustical analysis, recordings were transferred to Avisoft SASLab Pro (Version 4.40) and a fast Fourier transformation (FFT) was conducted. Spectrograms were generated with a FFT-length of 1024 points and a time window overlap of 75% (100% Frame, Hamming window). The spectrogram was produced at a frequency resolution of 488 Hz and a time resolution of 1 ms. A lower cut-off frequency of 15 kHz was used to reduce background noise outside the relevant frequency band to 0 dB. Call detection was provided by an automatic threshold-based algorithm and a hold-time mechanism (hold time: 0.01 s). An experienced user checked the accuracy of call detection, to assess concordance between automated and observational detection. Parameters analyzed for each test day included number of calls, duration of calls, and quantitative analyses of sound frequencies measured in terms of frequency and amplitude at the maximum of the spectrum.

#### Qualitative analysis

Qualitative analysis of ultrasonic calls was carried out for male pups only for consistency with our previous data. Waveform patterns of calls emitted by each pups were examined. Briefly, every USVs emitted at pnd 8 was classified in nine distinct categories, based on internal pitch changes, lengths and shapes, using our published categorization [22, 51].

Classification of USVs included nine waveform patterns described below: 1) *Complex* calls displayed one component containing two or more directional changes in pitch, each ≥6.25 kHz; 2) *Two-component* calls consisted of two components: a main call (flat or downward) with an additional punctuated component towards the end; 3) *Upward*-modulated calls exhibited a continuous increase in pitch that was ≥12.5 kHz, with a terminal dominant frequency at least 6.25 kHz more than the pitch at the beginning of the vocalization; 4) *Downward*-modulated calls exhibited a continuous decrease in pitch that was ≥12.5 kHz, with a frequency at least 6.25 kHz less than the pitch at the beginning of the vocalization; 5) *Chevron* calls resembled an ‘inverted-U’, which was identified by a continuous increase in pitch ≥12.5 kHz followed by a decrease that was ≥6.25 kHz; 6) *Short* calls were punctuated and shorter than 5 ms; 7) *Composite* calls were formed by two harmonically independent components, emitted simultaneously; 8) *Frequency steps* were instantaneous frequency changes appearing as a vertically discontinuous “step” on a spectrogram, but with no interruption in time; 9) *Flat* calls displayed a constant beginning and the ending of the pitch frequency remained constant (≤3 kHz of each other).

Call category data were subjected to two different analyses: a) treatment dependent effects on the frequency of vocalizations for each category emitted by each subject at pnd 8; b) treatment-dependent effects on the probability of producing calls from each of the nine categories of USV, as described below under Statistical analysis.

#### Somatic growth and righting reflex

One female and one male pup from each Veh- and CPF-treated litter were assessed for motor and somatic growth from postnatal days 4 to 12, as previously described [22, 52]. Body and tail length were also measured. The righting reflex was assessed by placing the pup on its back over a flat surface: the time needed to return to the natural position (all four paws on the floor) was measured using a stopwatch. The reflex was tested once in each day of assessment with a cut-off latency of 60 seconds.

#### Spontaneous movements

Concomitant with the USV recording on pnd 4, 6, 8 and 12, the spontaneous movements of the pups were also assessed. Frequency and duration of each behavioral item were analyzed by using NOLDUS OBSERVER software V 10 XT (Noldus Information Technology, Wageningen, NL, USA) to score the videotapes. In accordance with previous studies focused on neonatal rodent behavior [53–56], the following behavioral patterns were scored: locomotion (general translocation of the body of at least 1 cm in the glass container), immobility (no visible movement of the animal when placed with all the four paws on the floor), head rising (a single rising of the head up and forward), head shaking (a single lateral displacement of the head), face washing (forepaws moving back and forth from the ears to the snout and mouth), wall climbing (alternating forelimb placing movements on the wall of the container), pivoting (locomotor activity involving the front limbs alone and resulting in laterally directed movements), and curling (roll, vigorous side-to-side rolling movements while on the back; curl, a convex arching of back while on side or back, bringing head in a closer opposition to hump/hindlimb region). On the basis of previous studies [57–59] showing that in typically developing rodents calling rates are higher in time periods where animals show locomotion, thus suggesting a positive relationship between calling and motor behavior, we also analyzed whether occurrence of spontaneous movements was correlated with ultrasound emission.

### Assessment of adult behavior

#### Female-female and male-female social interaction

Litters were weaned on postnatal pnd 21 and littermates of the same sex and treatment were housed in polycarbonate cages (33x13x14 cm) and provided drinking water and a complete pellet diet (Altromin, Spezialfutter GmbH & Co. Germany) ad libitum. At adulthood one male and one female coming from Veh and CPF litter, and not previously subjected to neonatal behavior testing, underwent behavioral assessment from postnatal day 70 to 90. Females and males underwent female-female and male-female social interaction tests, respectively. Both sexes were also assessed for expression of repetitive grooming, as BTBR mice show characteristic high frequency of this behavior [18].

Social behavior tests were conducted in a novel cage of the same dimensions of the home cage with fresh bedding for male-female interactions and in the standard home cage of resident female for female-female social interactions; the experiments were made under red light and videorecorded and subsequently analyzed with NOLDUS OBSERVER software V 10XT by an observer blind to the treatment received by each mouse.

In male-female social interactions (n = 9 Veh; n = 9 CPF), the stimulus female mice (previously maintained in social groups of three females per cage) were introduced in the novel cage matched to the subject mice by age, body weight, and strain. On the day of male-female testing, the vaginal estrus condition of each female was assessed as previously described [60]. Only females in estrus were selected for the test. In female-female social interactions (n = 11 Veh; n = 10 CPF), an unfamiliar female mouse of the same strain was placed into the home cage of an isolated female test mouse coming from either Veh or CPF litters who had resided in the cage for the previous 5 days, for a 3-min session. In both tests social investigation was assessed by recording frequency and duration of sniffing of total body, head and anogenital areas, respectively, expressed by the experimental male or resident experimental female. USVs, normally emitted by male mice in presence of a sexually-receptive female [61] and emitted by resident female towards an intruder female, were also recorded with the ultrasonic microphone described previously, mounted 20 cm above the cage, to record the session for subsequent scoring of USV parameters. Vocalizations were recorded using Avisoft Recorder software version 3.2 at the same settings described above to record number of calls during the 3 min of the social interaction.

About 24 hrs after the social interaction tests, experimental male and female mice from both Veh and CPF litters (males, n = 9 Veh; n = 10 CPF; females, n = 10 Veh; n = 10 CPF) were scored for spontaneous grooming behaviors when placed individually in a clean, empty mouse cage without bedding [18, 21]. Each mouse was given a 10 min habituation period in the empty cage and then cumulative time spent grooming all body regions during a 10 min session was measured by using a stopwatch.

### Statistical analysis

Reproductive performance data was analyzed by analysis of variance (ANOVA) with treatment as between-subject fixed factor (2 levels).

A mixed-model ANOVA with repeated measures was performed to analyze treatment-dependent effects on neonatal USVs, spontaneous movement responses and developmental milestones, with treatment (Veh vs CPF) as factor and postnatal days and sex as repeated measures. The probability of vocalizations within treatment was calculated only for males at pnd 8 as number of calls in each category for each subject/total number of calls analyzed in each subject and standardized by angular transformation.

A mixed-model ANOVA with repeated measures was used to analyze sniffing behavior and number of USVs emitted with treatment as between-subject factor and either sniffing of different body areas (anogenital, body or nose to nose) or USVs displayed or emitted in each of the 3 minutes as repeated measures. A factorial ANOVA with treatment and sex as between subject factors was used to detect treatment differences in the amount of repetitive self-grooming and body weight at weaning and adult age.

Multiple regression analysis was performed to evaluate the correlation between neonatal motor responses (pivoting, head shaking and curling) and the ultrasonic vocalization rate, taking into account treatment, sex, postnatal day and their interactions (dummy variables in the regression model).

Post-hoc comparisons were carried out using Tukey HSD Test only when a significant F-value was determined. For all comparisons, significance was set at p = 0.05.

## Results

### Neonatal stage

#### Reproductive parameters

CPF administration to pregnant females did not induce any sign of systemic toxicity or impairment of weight gain during pregnancy. Gestation length, litter size, sex ratio and neonatal mortality were not affected by prenatal treatment with CPF. The mean body weight of the offspring at birth was not affected by CPF treatment as well (Table 1).

**Table 1:** Reproductive performance data of BTBR females receiving 6 mg/kg/bw CPF or Veh from GD 14 to 17.

Treatment	Number of pups	Sex Ratio	Body Weight
Veh	6.93 ± 0.6	0.77 ± 0.1	1.68 ± 0.6
CPF	8.62 ± 0.9	0.89 ± 0.1	1.71 ± 0.9

Data are indicated as Mean ± SEM. Veh, *n* = 26; CPF, *n* = 23

#### Vocalizations emitted by pups

**Fig 1:**
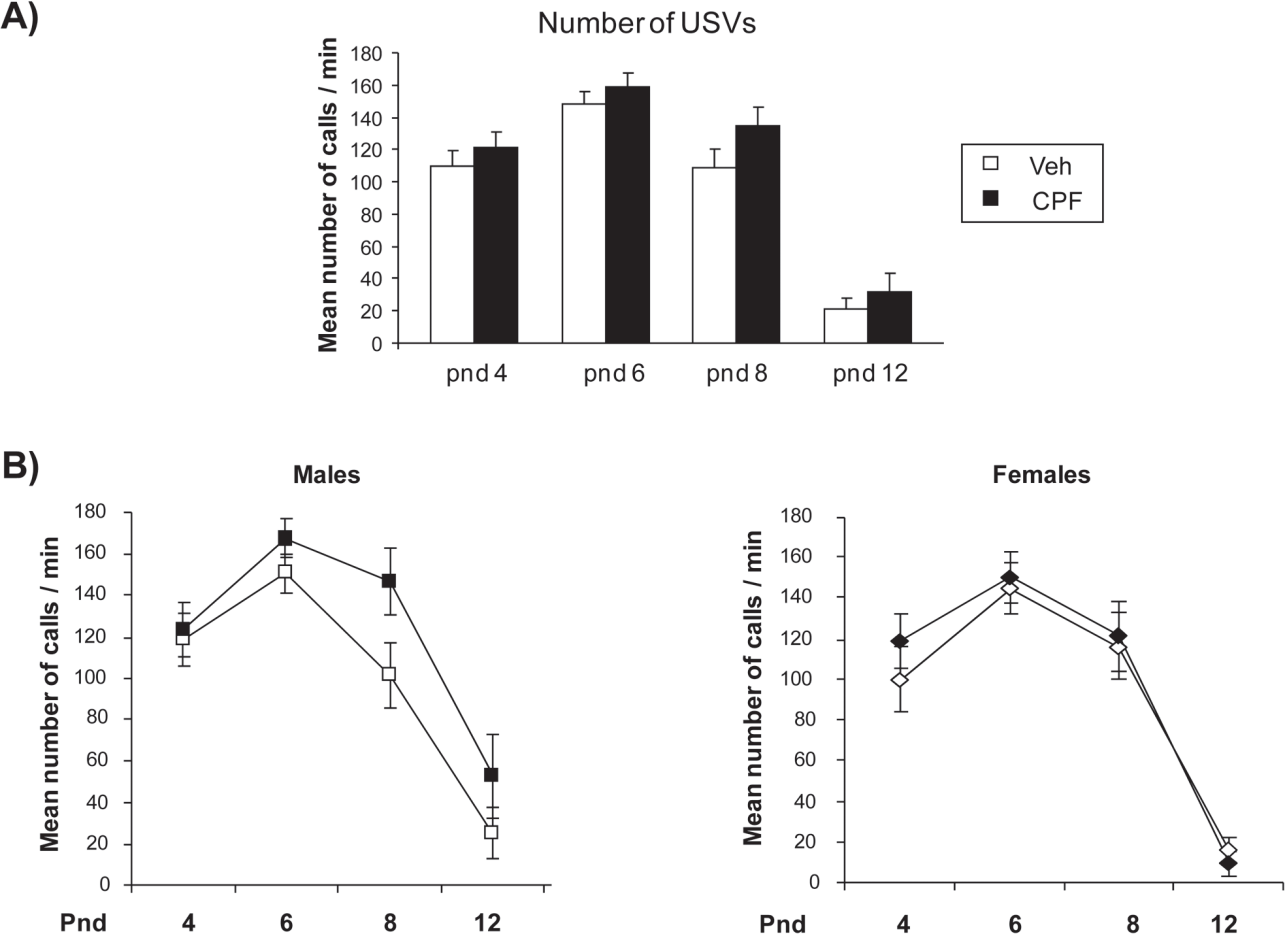
Ultrasonic vocalizations (USVs) in separated pups. A) Mean number of USVs emitted by CPF- and Veh-treated pups on postnatal day (pnd) 4, 6, 8 and 12 in response to three minutes of maternal separation. Data are expressed as mean number of calls/min ± SEM. Veh, *n* = 26; CPF, *n* = 23, including one male and one female deriving from each of the litters assigned to either treament. B) Mean number of USVs on pnd 4, 6, 8 and 12 emitted by CPF-treated and Veh pups of each sex. CPF tended to increase the rate of emission only in males. Data are expressed as mean number of calls/min ± SEM. Females: Veh, *n* = 13; CPF, *n* = 11; Males: Veh, *n* = 13, CPF, *n* = 12.

Analysis of USVs did not find a main effect of CPF treatment on calling rate. However CPF-treated pups tended to emit a higher number of ultrasounds [F (1, 47) = 3.19 p = 0.08] when isolated from the mother and siblings, a trend that seems more marked in male pups. CPF and Veh pups showed a similar temporal profile of emission, with peak of emission of USVs at pnd 6 [main effect of day, F (1, 3) = 75.530 p < 0.001]. As for duration, peak of frequency and peak of amplitude of calls emitted by pups neither a main effect of CPF nor a CPF interaction with postnatal days or sex was found (data not shown). The mean rate of calling at each day of assessment emitted by BTBR-Veh pups of both sexes was comparable to that reported in [22] (Fig. 1).

#### Classification of USVs into distinct categories

**Fig 2:**
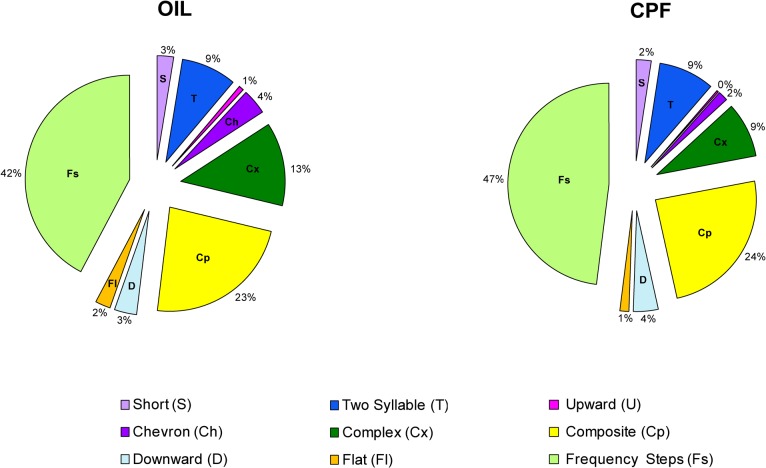
Pie graphs showing the percentages of the different call categories emitted by CPF- and Veh-treated males at postnatal day 8. Percentages were calculated in each treatment group as number of calls in each categories for each subject/total number of calls analyzed in each subject.

As for the evaluation of treatment-dependent variation on frequency of calls in males at pnd 8, no treatment effect was found [F (1, 22) = 0.273, p = 0.6065] (data not shown). Proportions of calls within each category are shown in Fig. 2. Accordingly to [22], both BTBR Veh- and CPF-treated mice, independently from the treatment received, emitted the spectrum of call categories previously described for the BTBR strain, with higher production of frequency steps, composite, two-syllable, along with lower production of short, chevron, flat, upward and downward. Both CPF- and Veh- treated pups showed a vocal repertoire characterized by 40% of frequency steps and 24% of composite, while CPF emitted 9% of complex and Veh 13% (Fig. 2).

#### Somatic growth and righting reflex

**Fig 3:**
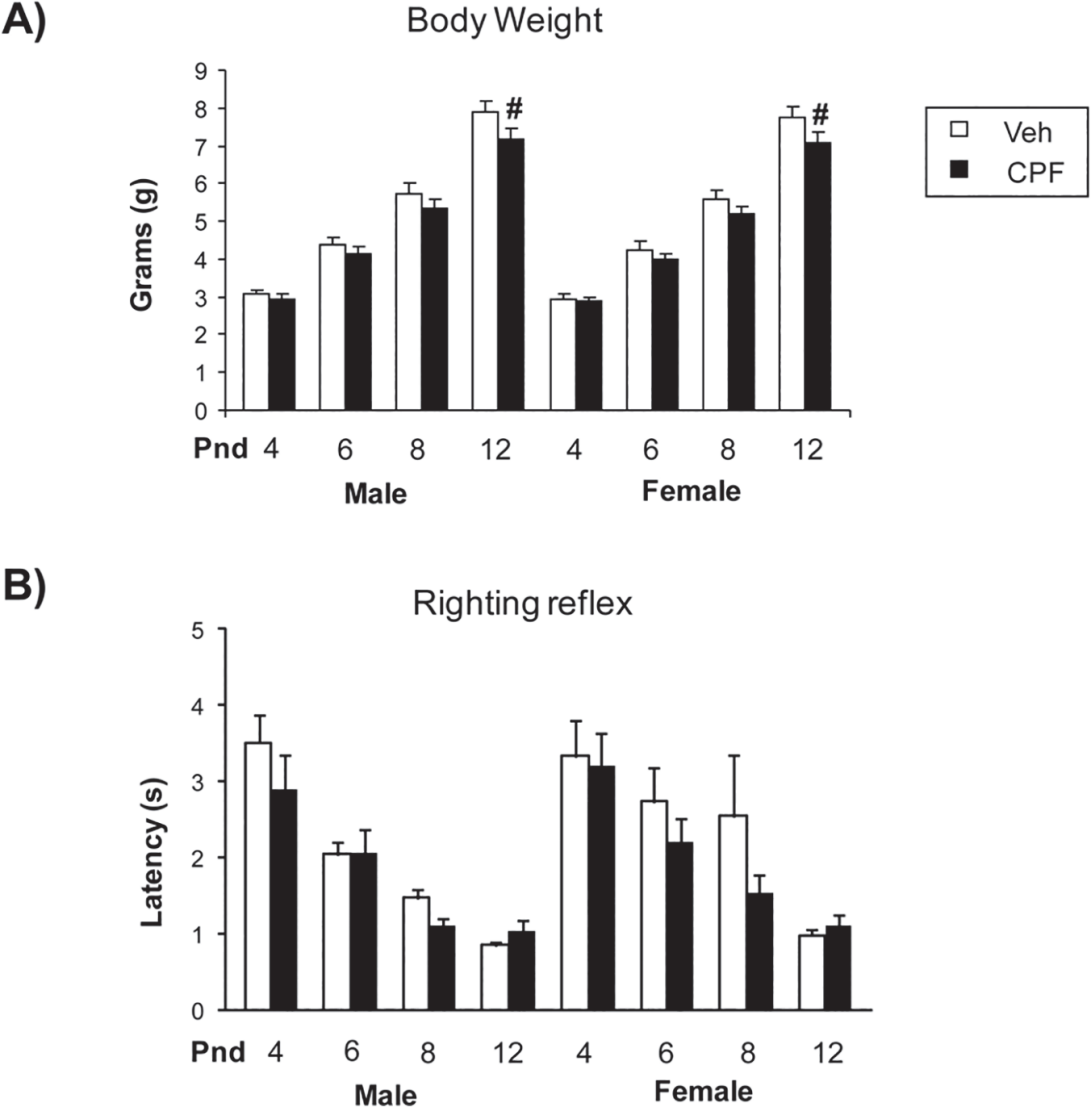
Somatic growth and righting reflex in pups. A) Analysis of body weight gain in BTBR pups at different postnatal days of testing. A significant postnatal days x treatment interaction was found, with CPF-treated pups weighing less than Veh controls. CPF treatment delayed the body weight growth of treated pups of both sexes on pnd 12 (#p < 0.05 after post hoc comparisons). B) Analysis of righting reflex latencies revealed no significant CPF effect in BTBR pups at different postnatal days of testing. Females Veh, *n* = 13; CPF, *n* = 11; Males Veh, *n* = 13, CPF, *n* = 12.

A significant postnatal days x treatment interaction was found for body weight [F (1, 66) = 3.04, p = 0.03], with CPF-treated pups weighing less than Veh controls. Specifically, CPF-treated pups showed significant lower body weight than Veh pups on pnd 12 (p < 0.05 after post hoc comparisons). The effect of CPF on body weight just missed significance on day 21 [F (1,23) = 27.204, p = 0.06], while at adulthood (70 days) this trend was no longer evident [F (1,18) = 12.853, p = 0.16]. As for body temperature, body length, tail length and righting reflex neither a main effect of CPF nor a CPF interaction with treatment, sex or day was found. The latency of righting reflex at each days of assessment (pnd 4, 6, 8 and 12) displayed by Veh pups of both sexes was comparable to that reported in [22] (Fig. 3).

#### Analysis of spontaneous movements

**Fig 4:**
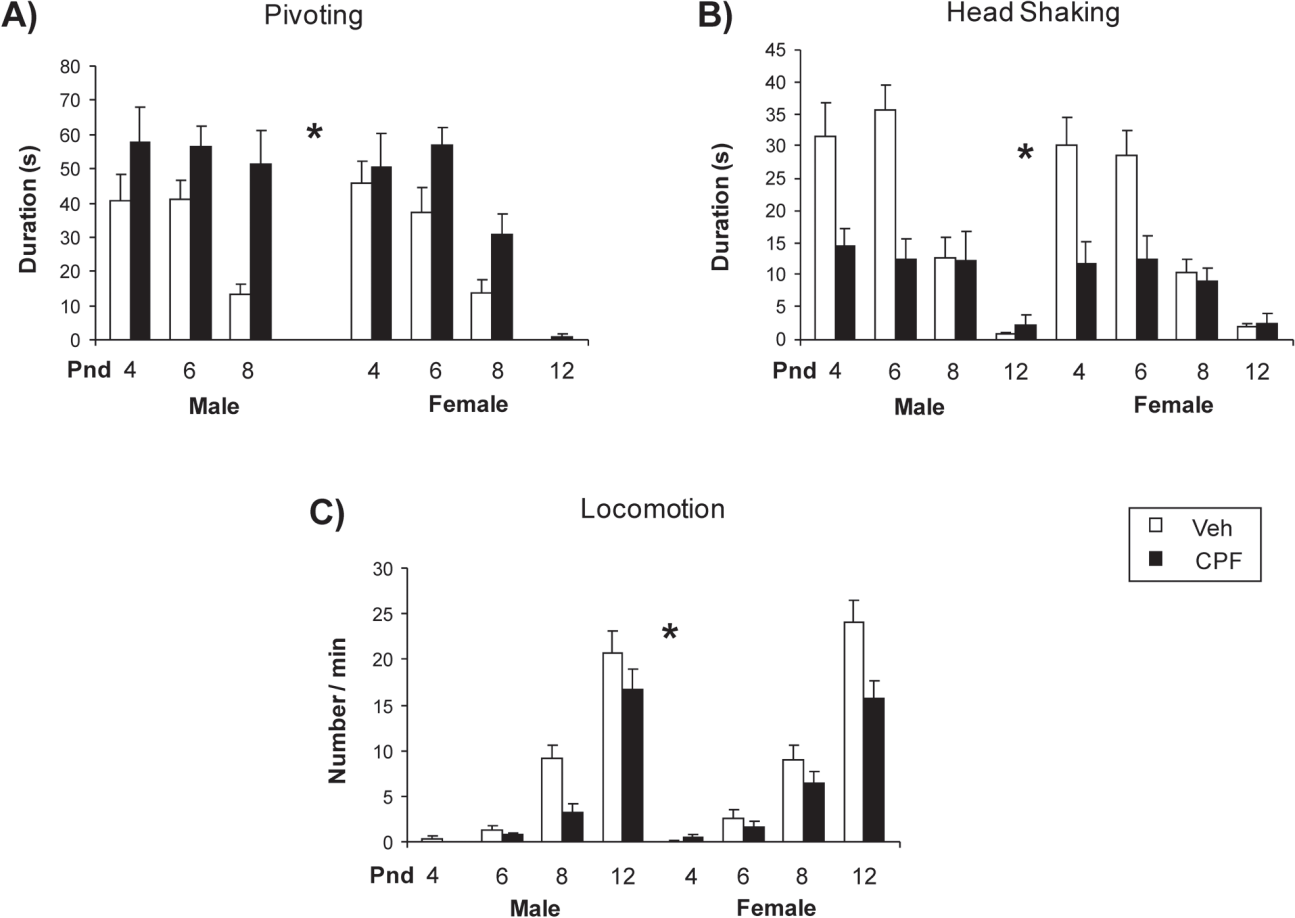
Duration and frequency of behavioral patterns shown by CPF-treated and Veh pups on pnd 4, 6, 8 and 12 during a 3-min session. A) Total duration of Pivoting, B) Head shaking and C) frequency of locomotion episodes. A main effect of CPF treatment was found for the three behavioral responses (p < 0.05; See Results section). Data are expressed as mean ± SEM. Females: Veh, *n* = 13; CPF, *n* = 11; Males: Veh, *n* = 13, CPF, *n* = 11.

The analysis of different motor indexes (Fig. 4) indicated a main effect of CPF on some of these indexes in both sexes. Duration of pivoting behavior evidenced a significant main effect of treatment with CPF treated pups exhibiting higher duration of pivoting than Veh pups at all days of assessment [F(1, 22) = 15.51, p < 0.001]. A similar profile was evident for frequency of pivoting behavior but the treatment effect just missed the statistical significance [F (1, 22) = 3.54, p = 0.07]. A main effect of the treatment was found for head shaking [frequency: F (1, 22) = 27.15, p < 0.01; duration: F (1, 22) = 24.057, p < 0.01], with CPF pups showing significantly less head shaking than Veh pups across all days of testing. ANOVA performed on frequency of locomotion episodes detected a main effect of CPF treatment across the four days of observation [F (1, 22) = 6.21, p < 0.05] with CPF pups showing lower frequency of locomotion in comparison to Veh pups. Post hoc comparisons performed on the interaction sex x postnatal days x treatment [F (3, 66) = 2,879, p = 0.0425] indicated that CPF female pups were less active than Veh females on pnd 12 (p < 0.05), while CPF males were less active than Veh on both pnd 8 and 12 (p < 0.05). No differences between treatments were observed for head rising, face washing, wall climbing, curling and immobility behaviors (data not shown).

Multiple regression analysis showed that, when taking into account treatment, sex, postnatal day and their interactions, the duration of pivoting and head shaking behaviors were positively related to the mean number of USVs emitted per minute. Specifically, for any one second increase in the duration of pivoting and head shaking behavior, the mean number of USVs/min increased of about one (pivoting: regression coefficient = 0.95, 95% CI = 0.62 to 1.27, p < 0.001; head shaking: regression coefficient = 1.03, 95% CI = 0.45 to 1.62, p = 0.001). The duration of curling was positively related to the mean number of USV/min, but this effect was not significant (regression coefficient = 1.29, 95% CI = -0.35 to 2.92, p = 0.121).

### Adult social interactions

#### Male-female interactions

**Fig 5:**
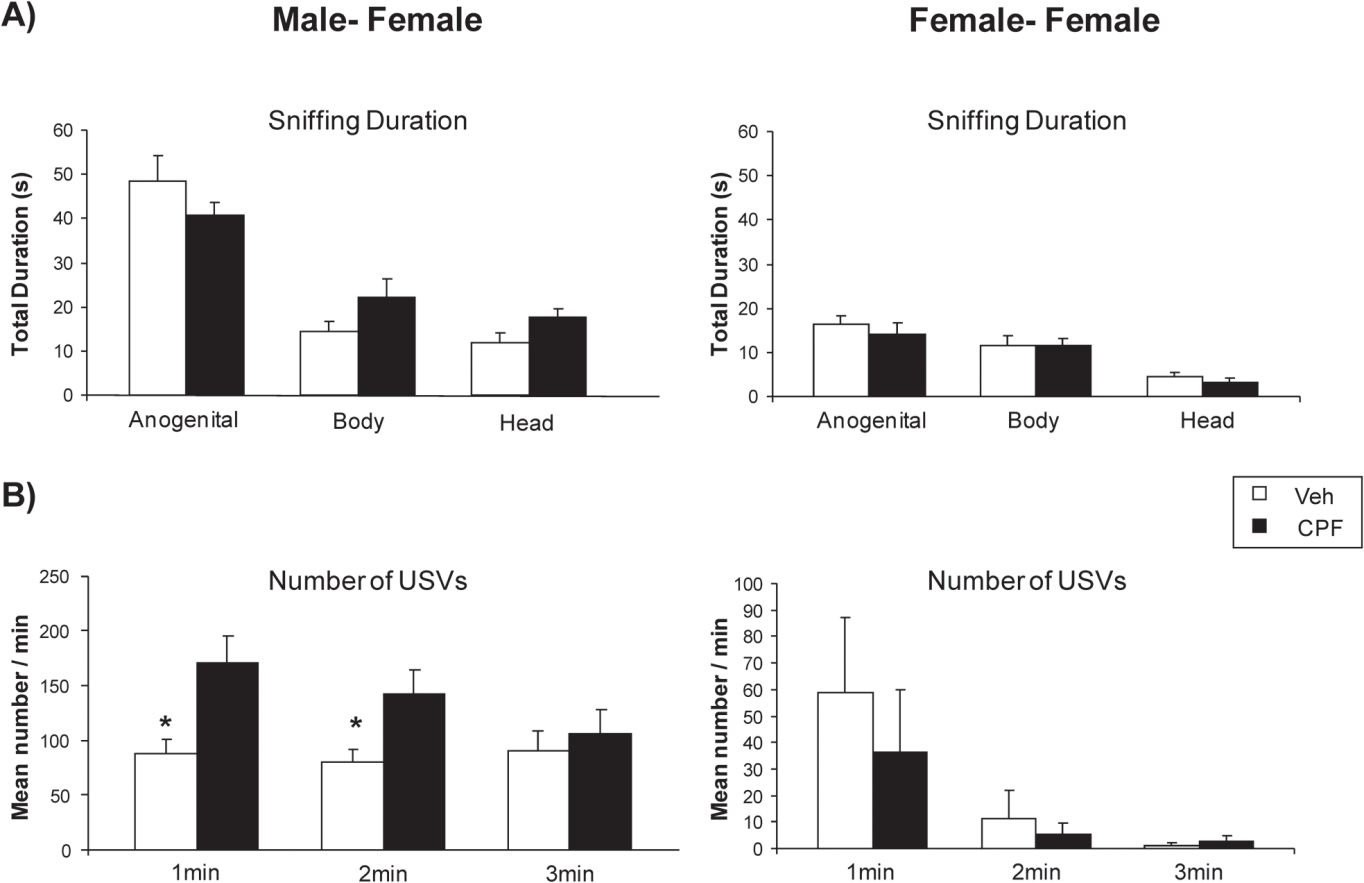
Adult social interaction shown by CPF- and Veh-treated mice of both sexes at adulthood. Left panel—Male- Female social interaction: A) Sniffing duration, B) number of USVs emitted during 3-min of direct interaction between male and a sexually-receptive female. Right panel—Female-Female social interaction: A) Sniffing duration, B) number of ultrasonic vocalizations emitted during 3-min of direct interaction between a resident and intruder female. Anogenital, Body and Head refer to the areas of the social partner's body investigated by the experimental subject. Data are expressed as mean ± SEM. *p < 0.05 after post hoc comparisons. Females: Veh, *n* = 11; CPF, *n* = 10; Males: Veh, *n* = 9, CPF, *n* = 9.

In the first phases of the social encounter between a male and a receptive female partner, the area of interest is typically the anogenital region of the female, which is investigated significantly more than the head and body areas. Scattoni and coworkers found that BTBR male mice display an atypical pattern of social investigation, as they sniffed the anogenital area of the estrus BTBR female partner significantly less as compared to C57BL/6J mice [Tukey’s HSD test: p < 0.001 for anogenital sniffing frequency and duration of C57BL/6J versus BTBR]. Here we found that the total duration of social investigation was not affected by CPF treatment (Fig. 5, left panel), but ANOVA yielded a significant interaction treatment x body areas [duration, F (2, 32) = 4.27, p = 0.02; frequency, F (2, 32) = 3.41, p = 0.04] with CPF treated males sniffing more frequently the body area than Veh males (Tukey’s HSD test: p < 0.05 for body sniffing frequency of CPF vs Veh). Furthermore CPF-exposed males emitted significantly more USVs when compared with Veh male mice [F (1, 16) = 13.67, p < 0.01] in response to the female presence during the 3-min of male-female social interaction. Post hoc comparisons performed on the significant treatment x min interaction showed that CPF effect was significant on the first and the second minute of the social encounter (Tukey’s HSD test for CPF vs Veh: first and second minutes, p < 0.05). USV emission rate in Veh BTBR mice of the present study was comparable to that observed in our previous paper where BTBR mice emitted lower number of USVs than C57BL/6J mice in response to a sexually receptive female. No significant effects were found for behavioral parameters used to assess general explorative activity, including rearing, digging and wall rearing (data not shown).

#### Female-female interactions

As shown in Fig. 5 (right panel), no significant CPF effect was identified as for social sniffing response towards an intruder female of the same strain [duration, F (1, 19) = 0.93 ns, frequency, F(1, 19) = 0.99 ns]. Analysis of USV emission showed no CPF effect across the three minutes of female-female social interaction test [F (1, 19) = 0.29 ns]. The values observed in Veh females as for social investigation and USV emission were comparable to our previous study [23].

#### Repetitive self-grooming

Analysis of self-grooming did not reveal either a main CPF effect on the levels of self-grooming in a 10-min session conducted in an empty cage [F (1,35) = 1.06 ns] or interaction between CPF treatment and sex. Specifically, the mean time expressed in seconds (± SEM) spent in self-grooming/ min was 30.71 ± 8.53 for Veh mice (n = 19), and 46.95 ± 13.07 for CPF mice (n = 20). The apparent increased proportion of time spent in self-grooming by CPF-treated mice was not significant possibly due to the high variability of the treated group. The frequency of grooming observed in this study is comparable to previous studies performed in the BTBR strain.

## Discussion

In this study we described several effects of the gestational CPF exposure in the BTBR mouse strain. Our findings highlight a specific vulnerability of BTBR mice to CPF as for early motor development, an effect not previously evidenced in other mouse strains. We show here that prenatal CPF exposure significantly modifies spontaneous motor activity in BTBR pups, delaying their motor development and further enhancing the abnormally high vocalization rates. The behavioral evaluation performed in both sexes at adulthood does not support the hypothesis that CPF worsens the atypical behavioral phenotype of BTBR mice, though the effects in males suggest a moderate amplification of the peculiar investigative behavior displayed during socio-sexual interaction in this rodent model of idiopathic autism [23].

The main effects of CPF exposure concern early motor development, in the absence of significant maternal or systemic toxicity. By means of longitudinal evaluation of spontaneous locomotion, scored during USV recording, we found that CPF exposed pups of both sexes exhibited longer duration of pivoting behavior which persisted up to postnatal day 8. During the first days of life in newborn mouse pups the hind limbs do not support the movements of the forelimbs in a coordinated way, and the pelvis remains anchored to the ground. Uncoordinated forelimb movements produce a circular movement defined as pivoting behavior [62]; in typically developing mouse lines, this response is higher during the first week, and it is progressively replaced by a fine-coordinated locomotion associated with development of the forelimbs. In CPF-treated BTBR mice, the persistence of pivoting behavior until pnd 8 is paralleled by the reduction of head-shaking behavior that requires a fine coordination of the neck muscles; this reflex is significantly reduced in both CPF males and females at each day considered.

The prerequisite of fine-coordinated locomotion is the sustained antigravity support of the head by the neck muscles and of the trunk by the extensor of the limbs [62]: thus CPF appears to delay the acquisition of this integrated reflexes, with the persistence of immature behavioral patterns at the expenses of adult-like locomotion that was also significantly reduced up to pnd 12. Though the mechanisms implicated in CPF effects are out of the scope of the present study, it is worth mentioning that central monoaminergic—i.e., dopaminergic (DA), serotoninergic (5-HT), and noradrenergic (NA)—pathways play a major role in the maturation of spinal locomotor networks in rats and mice. Notably, mice lacking the enzyme degrading 5-HT and presenting perinatal 5-HT excess have delayed motor development in comparison to control mice, in terms of retarded coordination of fore- and hind limbs [63]. Persisting effects of developmental CPF have been found on monoaminergic pathways and at doses below those which cause appreciable brain cholinesterase inhibition. Specifically, the brain 5-HT systems are among the most sensitive to CPF effects: for exposure on GD 17–20 in rats at a dose comparable to that used in the present study, Aldridge and coworkers found markedly large increases in 5-HT receptor and transporter expression in several brain areas at the juvenile stage [64]. These same authors hypothesized that gestational CPF exposure generates miswiring of 5-HT circuits in the perinatal phase that may be responsible for the later dysfunction of the 5-HT systems with manifest effects on behavior at the adult age [39]. Interestingly, BTBR mice also show abnormalities of 5-HT neurotransmission, as they present reduced density of 5-HT transporter and heightened responsiveness of postsynaptic 5-HT receptors in the hippocampus, the only region so far analyzed [65]. It is possible that the constitutive altered regulation of central 5-HT neurotransmission renders the BTBR mice particularly vulnerable to the effects of CPF on the developing 5-HT systems, to the point that motor effects became evident as early as the first week of life, at variance with what found in the out bred CD1 strain. Further studies are needed to address this hypothesis, as well as to investigate other neural pathways sensitive to CPF effects that may participate in the delayed motor development of BTBR pups, included the dopaminergic and the cholinergic ones.

In spite of the general motor delay, the maturation of righting reflex and curling behavior (i.e. the side to side rolling movement while on the back aimed at righting) were not affected by CPF exposure. Both responses are strongly associated with each other, and require the integrity of muscular and motor function for the adequate acquisition of symmetrical coordination between the left and right sides of the body [66]. Righting reflex appears soon after birth in rodents, earlier than pivoting behavior, and its correct performance likely depends on the integration between vestibular system and the motor system executing it [62, 67]. However some of the connections of the vestibular structure with other brain areas develop only in the first days of life in rodents, and this could in part explain the relative insensitivity of the righting reflex to CPF effects. Finally, the assessment of developmental milestones also shows that CPF delayed body growth in this mouse strain, with reduced values of body weight on pnd 12 in both sexes, an effect fully recovered by adulthood.

Altogether, delay in reflexes requiring a fine control of the motor coordination and persistence of immature motor milestones associated with the reduction of locomotion episodes displayed by CPF pups, were also shown by other animal models of ASD, such as Mecp2-null, Foxp2 and Reeler mice and out bred mice undergoing gestational exposure to valproate [47, 55, 68, 69]. Thus, fetal CPF exposure causes in the BTBR strain early neuromotor abnormalities that appear as a hallmark of murine models of autism. From a translational perspective these results are in partial agreement with a human epidemiological study evidencing decreased somatic growth at birth in neonates associated with cord plasma CPF levels [70]. Furthermore, many epidemiological findings suggest that there is an association between maternal OPs exposure and neurodevelopment in children, in particular in the CHAMACOS cohort study the presence of OP metabolite dialkylphosphate (DAP) in maternal urine or blood was associated with impaired reflex functioning in infants after pnd 3 [71]. More importantly, several studies suggest that anomalies in specific motor milestones (such as lying, sitting and walking) may serve as early markers for ASD diagnosis, and one possible explanation of these motor alterations is related to Purkinje cell disruption in the cerebellum [72]. Detection of subtle abnormalities in infants' general movements may be indicative of later neurological and neuropsychiatric conditions [73, 74], in particular infants with ASD display atypical movements and reduced motor coordination which may precede social-communication deficits within the first year of life [75].

We have previously shown that BTBR pups have higher emission of ultrasonic vocalizations and restricted repertoire of the type of calls emitted when compared to other inbred strains [22]. CPF exposure does not significantly modulate this strain-specific characteristic, though CPF-treated BTBR pups tended to emit even more calls than Veh-treated pups, a trend more evident in males at pnd 8. Thus, prenatal CPF exposure only tended to amplify a distinctive sign of the early development of BTBR mice, namely the high vocalization rates characteristic of this strain, and failed to influence the characteristic vocal repertoire of vocalizations recorded in the BTBR strain. Our results confirm data on the limited vocal repertoire of BTBR [22], as both CPF- and Veh-treated males displayed the same categories of vocalizations (*frequency steps*, *composite* and *complex*) previously observed in unhandled BTBR pups.

The developmental profile of CPF-treated BTBR mice is different from that we previously observed in the out bred CD-1 strain gestationally exposed to CPF with the same dose and schedule of the present study. In CD-1 mice, prenatal CPF exerted a clear depressive effect on USVs with decreased number and duration at pnd 10 and higher peak of frequency of the calls. Additionally, in the CD-1 strain the altered profile of ultrasound emission was accompanied by significant reduction in pivoting frequency associated with higher duration of immobility [46]. This strain-related difference supports the complex interaction occurring between gene background and toxicants’ effect, as also shown by the paradoxical findings reported by Laviola and co-workers in new born *reeler* mice, where prenatal exposure to CPF oxon reverted some of the features of the pathological phenotype of this transgenic mouse strain while delaying acquisition of the righting reflex [76].

At adulthood CPF-associated alterations were found predominantly in males, while in social interactions between females we did not found any significant change in social investigation, differently from what evidenced in out bred mice [77]. Specifically, in BTBR males, CPF increased social sniffing in body and head areas and reduced sniffing in the anogenital area of sexually receptive-female, thus enhancing the shift from anogenital to body areas characteristic of the BTBR strain. Surprisingly, this particular shift in social investigation is accompanied by a significant increase in the rate of emission of ultrasonic vocalizations during the courtship that may be difficult to be interpreted if not considering it as a behavioral marker of arousal, already described in male mice exposed to CPF [43, 78].

In conclusion, our findings indicate that prenatal CPF exposure, at doses devoid of maternal or systemic toxicity, significantly affects early neurobehavioral maturation of BTBR mice, which appear to be influenced by this neurotoxicant to a larger extent than reported in the out bred CD-1 strain. We found very limited effects of prenatal CPF on adult behavior: it is possible that behavioral paradigms specifically designed as to reveal the occurrence of motor stereotypies, the deficit in social motivation and the lack of behavioral flexibility typical of the BTBR strain (i.e. the social approach or the social conditioned place preference) could evidence enhanced vulnerability to the developmental neurotoxicity of the OP insecticide at the adult stage too. In addition, there is indication of potential non-monotonic dose-response effects of OP insecticides on learning functions [79], as at the higher doses even a limited and transient enhancement of cholinergic neurotransmission might be paradoxically protective for the developing brain insofar that compensatory mechanisms are called upon. On these bases, we cannot exclude that a lower dose of CPF could have affected the adult BTBR phenotype to a greater extent; further experiments are in progress to verify this hypothesis.

To our knowledge this is the first attempt to analyze the effects of a widely diffused environmental chemical in a validated mouse model of ASD. Rodent models are useful tools to identify potential gene–environment interactions that might contribute to neurodevelopmental alterations and to test what gene–environment combinations produce the greatest neurobehavioral deficits. Therefore, future research in animals must consider how different factors may work in combination to affect brain development, in order to understand how genetic susceptibility and environmental chemicals interact to produce measureable deficits in brain and behavioral development. Derailment of early development trajectories induced by a commonly used insecticide in a model of idiopathic autism requires further experimental investigation applying a larger spectrum of behavioral tests.
